# Supplementary material for: A life course perspective on predictors of midlife socioeconomic status
Source: PLoS One. 2025 Sep 10;20(9):e0330130. doi: 10.1371/journal.pone.0330130 (PMC12422441; doi:10.1371/journal.pone.0330130)
Supplement: Attachment — Submitted filename: review .docx [file pone.0330130.s001.docx]

Review:

I read this paper with great interest, and believe it has the potential to become a widely referenced source on the important topic of life course influences on adult socio-economic status. I will mention some of the important aspects of the paper that make it likely to become a widely referenced source, followed by a few suggestions that might improve the impact of the study and the clarity of the manuscript.

1. This is a well conducted replication and extension of previous studies: It replicates main consistent findings in the literature, and extends these to a relatively large, new population, context, and age cohort, with a broad range of childhood SES backgrounds. In addition there are important new features here: e.g., the multiple time points for prediction to mid adulthood (early childhood, later childhood, early adulthood); the addition of a range of predictors that have not been widely examined (these typically show small direct effects, but are important mediators of earlier predictors); and, most importantly, the focus on indirect effects and mediation of established predictors.
2. This design allows for comparison of the relative effect sizes (both direct and indirect) of predictors. It will therefore be a useful reference for future studies with other cohorts.
3. Studies directly focused on prediction of mid-adult SES are relatively unusual (often SES is considered as a predictor or control variable in longitudinal designs rather than an outcome). As the effects of the “established” predictors appear to vary somewhat across populations, contexts, etc. it is important to add to the body of literature with additional projects with different populations, contexts, age cohorts etc. so that this variability of results can be interpreted and understood. The importance of replication in a research area which is largely observational is well established.
4. This design allows identification of important “intermediate” outcomes that may be more amenable to intervention and policy decisions than the established predictors (e.g. parental education, occupational status, childhood IQ).

There are some limitations in the present paper based on the data set, which are well identified and stated in the manuscript. Most important of these are the limitations of using a single sex (male) which was available from the original sample, and some limitations in the type of predictive measures which were available from the data set (as described in the paper).

My main concern regarding limitations is that moderation effects were not reported. In addition to mediation, moderation is likely to be a main source of variance in this type of analyses. The authors explain that it would require an entire additional manuscript and analysis to examine these, but I would argue that we only have “half the story” here. It is an extremely interesting and potentially useful “half story” and so will be of broad interest to readers, but from both conceptual and applied/policy perspectives, the moderation processes should be considered as well. Could there be an appendix, or a related/complementary paper made available from the data set?

A minor specific issue: Please explain why weighted loadings were used for occupational status and income (p.9, ln.19). This influenced the results, as stated, but we don’t know what the rationale was….

In sum, I would like to know more about the interaction effects (moderation) in this or a complementary report. However, I am very impressed with the information and design presented here. I think it has the potential to be widely read and replicated, as well as used for social and educational policy development.
